# Supplementary material for: DMRforPairs: identifying Differentially Methylated Regions between unique samples using array based methylation profiles
Source: BMC Bioinformatics. 2014 May 15;15:141. doi: 10.1186/1471-2105-15-141 (PMC4046028; doi:10.1186/1471-2105-15-141)
Supplement: Additional file 2 — DMRforPairs output for the comparison of A431-MCF7 and NA17018-NA17105. Please start from the HTML files in each folder. Available via the BMC Bioinformatics website. [file 1471-2105-15-141-S2.zip › 1394847754114233_MOESM2_ESM/A431_MCF7/figures/34088.pdf]

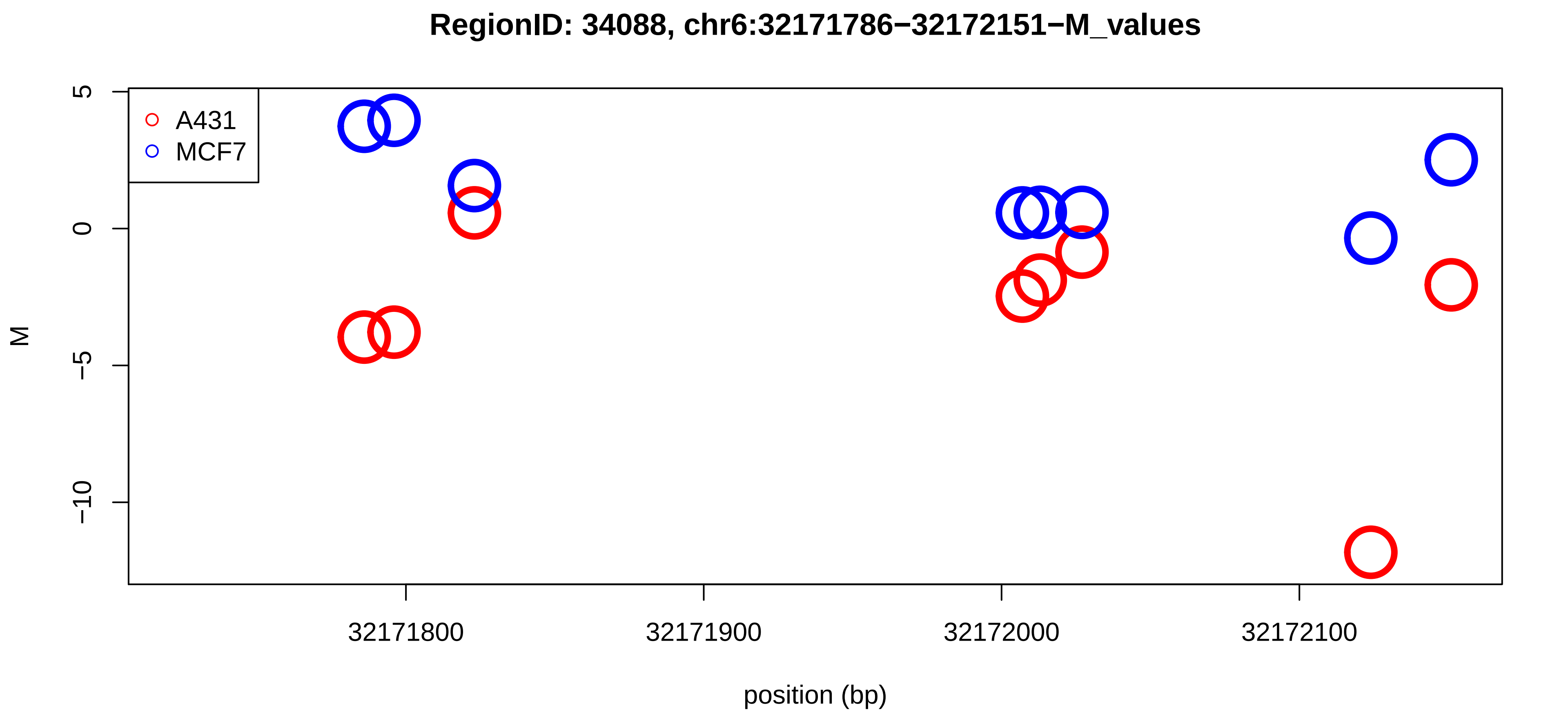
RegionID: 34088, chr6:32171786−32172151−M_values
A431
MCF7
M
5
0
−5
−10
32171800
32171900
32172000
32172100
position (bp)

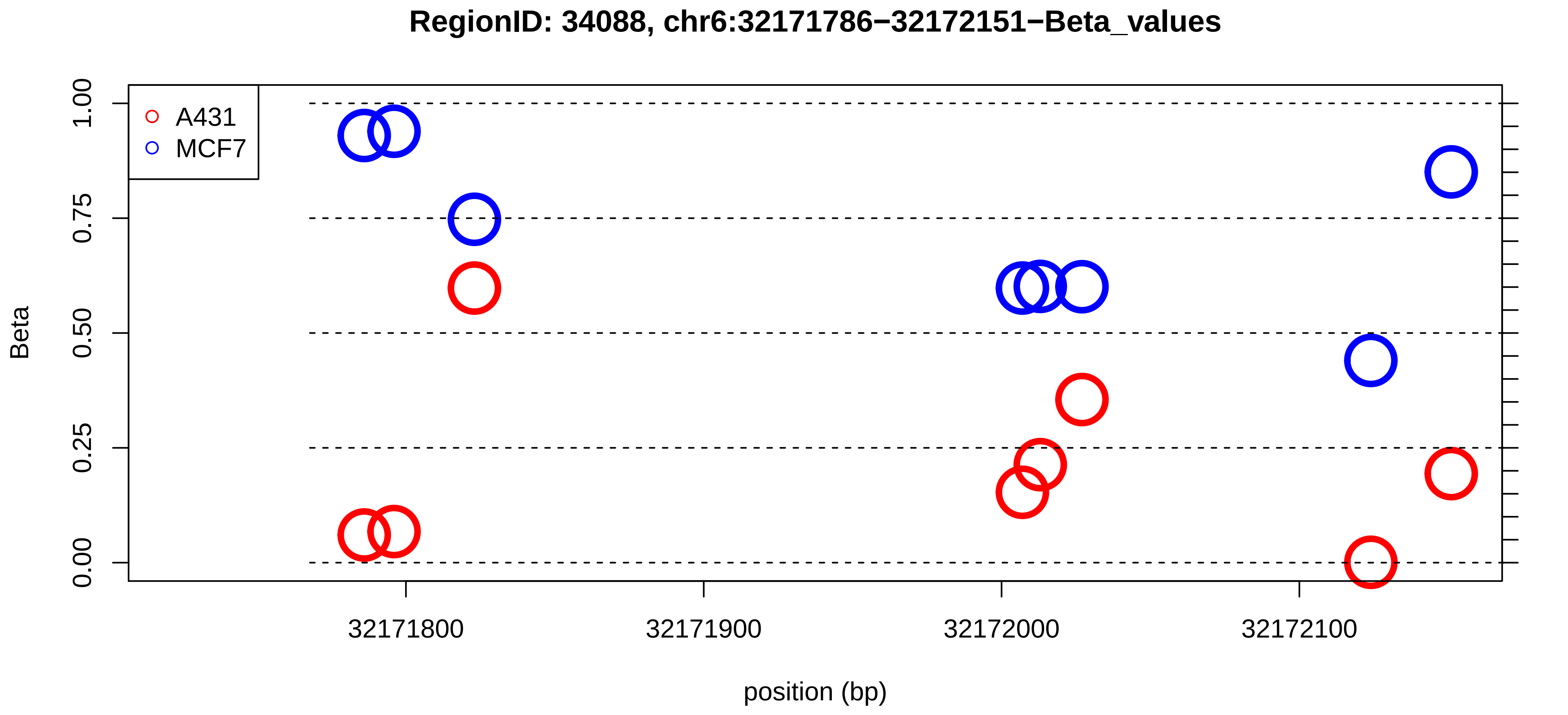
RegionID: 34088, chr6:32171786−32172151−Beta_values
A431
MCF7
Beta
1.00
0.75
0.50
0.25
0.00
32171800
32171900
32172000
32172100
position (bp)

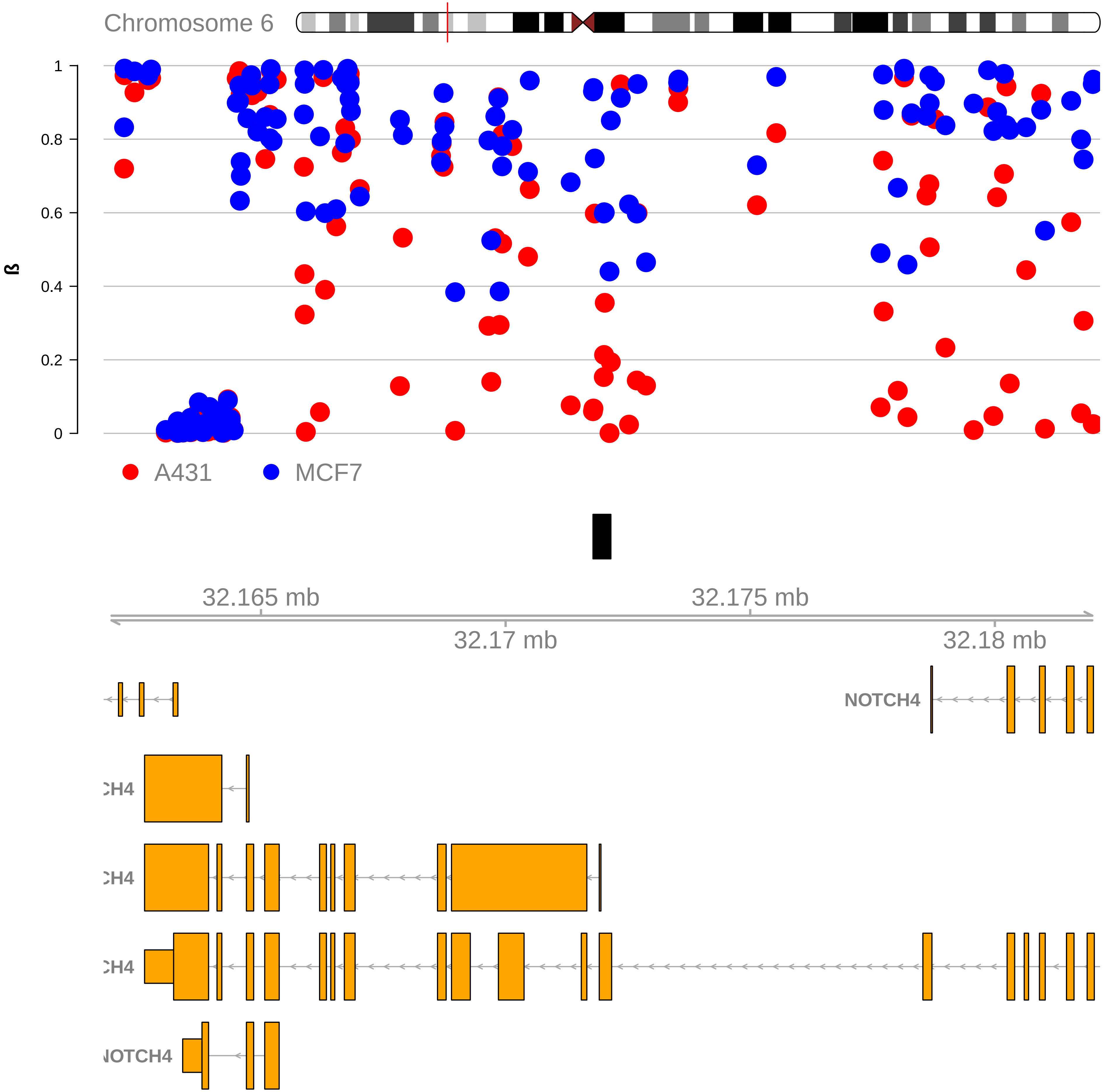

Chromosome 6
1
0.8
0.6
0.4
0.2
0
ß
A431
MCF7
32.165 mb
32.175 mb
32.17 mb
32.18 mb
NOTCH4
CH4
CH4
CH4
NOTCH4
